# Trends and inequalities in HIV testing uptake among pregnant women during antenatal care in Ghana: a decomposition analysis from 2008 to 2022

**DOI:** 10.1186/s41182-025-00897-0

**Published:** 2026-01-05

**Authors:** Amidu Alhassan, Patience Fakornam Doe, Yula Salifu, Asirifi Isaac Gunu, Joseph Lasong, Mustapha Amoadu

**Affiliations:** 1 Department of Adult Health, School of Nursing and Midwifery, College of Health and Allied Sciences, University of Cape Coast, Cape Coast, Ghana; 2 Department of Public Health, School of Nursing and Midwifery, College of Health and Allied Sciences, University of Cape Coast, Cape Coast, Ghana; 3 Biomedical and Clinical Research Centre, College of Health and Allied Sciences, University of Cape Coast, Cape Coast, Ghana; 4 Department of Population, Family and Reproductive Health, School of Public Health, University of Ghana, Legon, Ghana; 5 Department of Nursing, School of Biomedical Engineering and Allied Health Sciences, All Nations University, Koforidua, Ghana; 6 Department of Population and Reproductive Health, University for Development Studies, Tamale, Ghana

**Keywords:** HIV testing, Antenatal care, Inequalities, Pregnant women, Ghana

## Abstract

**Background:**

Human immunodeficiency virus (HIV) testing during antenatal care (ANC) is pivotal for prevention of mother-to-child transmission (PMTCT), facilitating early initiation of antiretroviral therapy, infant prophylaxis, and retention in care. While coverage has improved globally, inequalities threaten progress towards Sustainable Development Goal (SDG) 3.3 on ending the AIDS epidemic. This study examined trends and inequalities in HIV testing uptake among pregnant women during ANC in Ghana between 2008 and 2022.

**Methods:**

Data were drawn from the 2008, 2014, and 2022 Ghana Demographic and Health Surveys, comprising a pooled sample of 41,574 women aged 15–49 years who had given birth within two years preceding each survey. The World Health Organization’s Health Equity Assessment Toolkit was applied to estimate Differences (D), Ratios (R), Absolute Concentration Indices (ACI), Population Attributable Fractions (PAF), and Population Attributable Risks (PAR) across equity stratifiers which include ge, education, marital status, residence, region, and wealth.

**Results:**

The study found that the national coverage of HIV testing during ANC increased from 28.9% in 2008 to 61.0% in 2014 and 72.4% in 2022. Uptake improved among women with no education (18% in 2008 to 60% in 2022) and rural women (20–70%). Nonetheless, wealth quintile, uptake in 2022 ranged from 92.3% in the richest to 51.7% in the poorest; the ACI was 8.2% (95% CI 7.2–9.1) and PAF 27.5% (95% CI 27.4–27.5). Regional disparities were largest with Volta achieving 88.9% versus 32.8% in Savannah, with D rising from 39.7% in 2008 to 56.1% in 2022, while PAF fell from 70.1% (95% CI 69.8–70.3) to 22.7% (95% CI 22.7–22.8). Educational inequalities narrowed; PAF declined from 106.0% (95% CI 105.4–106.5) in 2008 to 34.8% (95% CI 34.8–34.9) in 2022. Age-related differences were negligible, with ACI 1.8% (95% CI − 0.6 to 4.2) in 2022.

**Conclusion:**

Ghana has achieved substantial expansion of ANC-based HIV testing over the past decade. However, pronounced regional and socioeconomic inequalities remain. Targeted, equity-oriented interventions focusing on northern regions and poorest households are essential to prevent avoidable paediatric HIV infections and to sustain progress towards SDG 3.3.

## Introduction

Human immunodeficiency virus (HIV) testing during antenatal care is a cornerstone of prevention of mother-to-child transmission (PMTCT). Globally, an estimated 40.8 million people were living with HIV in 2024 [1]. Additionally, 84% of pregnant women living with HIV received antiretroviral medicines to prevent vertical transmission globally [2]. Sub-Saharan Africa bears a disproportionate burden, with women and girls accounting for 45% of new infections in the region in 2024 [3]. Also, pooled analysis shows that around 73.9% of women in sub-Saharan Africa are tested for HIV during ANC [4], but large between-country and within-country inequalities persist. In Ghana, the 2022 Ghana Demographic and Health Survey (DHS) reported that almost three-quarters (72%) of women aged 15–49 who gave birth in the two years before the survey were tested for HIV during ANC or labour and received their results, yet overall testing remains uneven [5]. The same DHS showed that 2.2% of women aged 15–49 had ever been tested for HIV, and only 15% had been tested in the previous 12 months [5], with substantial variation by residence, region, education, and wealth quintile.

The importance of HIV testing in ANC extends beyond maternal health, as it directly influences child survival and broader population health outcomes [6]. Early detection allows mothers to commence antiretroviral therapy [7, 8]. Again, it also offers opportunities for counselling, disclosure, and linkage to care, enhancing adherence and retention across the HIV treatment cascade [9, 10]. Furthermore, universal testing at ANC visits strengthens health systems by normalising HIV services, reducing stigma, and contributing to national progress towards epidemic control [11, 12].

Multiple, intersecting determinants make ANC-based HIV testing a continuing concern among pregnant women. Recent evidence from across sub-Saharan Africa identifies lower uptake among rural residents and poorer, less-educated women, while higher uptake is associated with comprehensive HIV knowledge, employment, and higher wealth [4]. Programme factors, including testing availability, integration with other ANC services, and implementation of retesting policies during late pregnancy and the postpartum period, also shape coverage [11, 13, 14]. In Ghana, near-universal ANC contact co-exists with variable content and quality of services, implying missed opportunities for consistent HIV testing and retesting [15, 16]. Social barriers such as discriminatory attitudes towards people living with HIV further depress demand and completion of the testing cascade [17, 18].

Despite policy commitment and measurable gains, there is limited equity-focused national evidence that tracks trends and socioeconomic, geographic, and demographic inequalities in ANC-based HIV testing among pregnant women in Ghana over time. Existing analyses often report either overall testing among women or single-survey snapshots [19–21], leaving unanswered questions about how inequalities have evolved across regions, residence, education, and wealth, and how these gaps translate into missed PMTCT opportunities in routine practice. This evidence gap constrains targeted planning, efficient resource allocation, and the design of retesting strategies that prioritise underserved populations, thereby perpetuating avoidable infant infections and undermining community trust. This study addresses the gap by analysing trends and inequalities in HIV testing uptake during ANC in Ghana, with disaggregation by key equity stratifiers. Evidence from this analysis will inform Ghana Health Service planning, sharpen targeting of integrated ANC-HIV services, and support progress towards SDG 3.3 through equity-oriented PMTCT programming.

## Methods

### Study design and data source

This study adopted a cross-sectional design using nationally representative data from the Demographic and Health Surveys conducted in Ghana in 2008, 2014, and 2022, with a pooled sample of 41,574 women. The surveys were implemented by the Ghana Statistical Service (GSS) in collaboration with the Ghana Health Service (GHS) and ICF International. They generate high-quality data on maternal and child health, human immunodeficiency virus, and related population health indicators. A two-stage stratified sampling design was applied to ensure representativeness at national, regional, and rural–urban levels.

### Study population and eligibility

The study population comprised women aged 15–49 years who had given birth within the two years preceding each survey. Eligible respondents were those who reported attending ANC and were asked whether they were tested for HIV during ANC or labour and whether they received their results. Women with incomplete responses to these questions were excluded from the analysis.

### Outcome variable

The primary outcome was uptake of HIV testing during ANC or labour, defined as pregnant women who reported being tested for HIV and receiving their results. This binary outcome variable was coded as 1 (tested and received results) and 0 (not tested or did not receive results).

### Equity stratifiers

To examine inequalities, the study employed key equity stratifiers aligned with the World Health Organization’s Health Inequality Data Repository and the Health Inequality Assessment Toolkit (HEAT). These comprised socio-demographic characteristics, including age (seven groups, 15–49 years), educational attainment (no education, primary, secondary, higher), marital status (never married, married or living together, widowed, divorced, or separated), and place of residence (urban or rural). Economic status was assessed through the household wealth index, categorised into quintiles ranging from the poorest to the richest. In addition, geographic variation was captured using subnational regions, defined according to the administrative boundaries at the time of each survey.

### Statistical analysis

Weighted analyses were conducted to account for survey design, clustering, and sampling probabilities. Descriptive statistics summarised uptake rates across equity stratifiers and survey years. Trends in HIV testing uptake from 2008 to 2022 were assessed. To quantify inequalities, decomposition analyses were performed using measures recommended by WHO HEAT, including the Absolute Concentration Index (ACI), Difference (D), Population Attributable Fraction (PAF), Population Attributable Risk (PAR), and Ratio (R). These indices provide complementary assessments of the magnitude and direction of disparities across socioeconomic, demographic, and geographic subgroups. The Absolute Concentration Index is a complex, weighted measure for ordered dimensions that summarises the degree to which a favourable health indicator is concentrated among advantaged or disadvantaged subgroups, taking a value of zero under perfect equality and positive values when concentration favours advantaged groups [22]. Difference is a simple, unweighted absolute measure calculated as the difference between the estimates of the most advantaged and most disadvantaged subgroups, with larger absolute values indicating wider inequality. Ratio is a simple, unweighted relative measure expressed as the quotient of the estimate in the most advantaged subgroup divided by that in the most disadvantaged subgroup, where a value of one denotes equality. Population Attributable Risk is a complex, weighted absolute measure defined as the difference between the estimate of the reference subgroup and the national average, representing the absolute improvement that could be achieved if all subgroups attained the reference level. Population Attributable Fraction is the corresponding relative measure, calculated as the Population Attributable Risk divided by the national average and multiplied by 100, indicating the proportional gain in national coverage achievable under an equity scenario. All measures were generated within the WHO HEAT platform using survey-weighted data and analytical variance estimation procedures, following established World Health Organization guidance on health inequality monitoring [22–24]. Ninety-five percent confidence intervals (CIs) were estimated for all measures.

### Ethical considerations

The analysis was based on anonymised, publicly available data from the Ghana Demographic and Health Surveys (DHS). Ethical approval for data collection was obtained by the Ghana Statistical Service, Ghana Health Service, and ICF International, with informed consent secured from all participants. The DHS surveys were conducted in accordance with the Declaration of Helsinki. No further ethical clearance was required for this secondary analysis.

## Results

### Inequalities in HIV testing uptake among pregnant women during antenatal care in Ghana by region

**Fig. 1:**
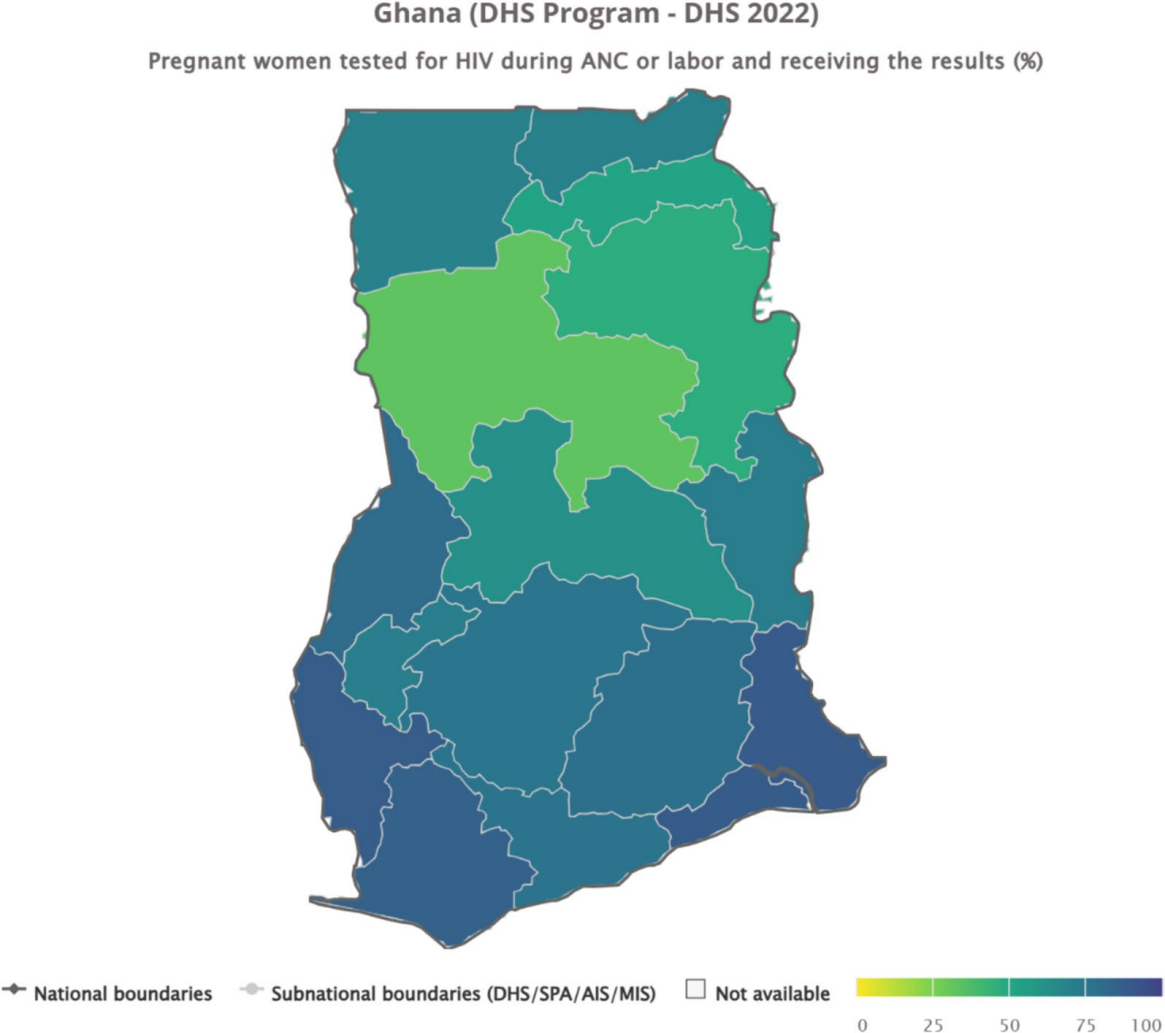
Inequalities in HIV testing uptake among pregnant women during antenatal care in Ghana by region

The data reveal wide regional disparities in the proportion of pregnant women tested for HIV during ANC or labour and who received their results in Ghana. Coverage was highest in the Volta (88.9%), Greater Accra (88.4%), Western North (87.9%), and Western (85.4%) regions, reflecting stronger health system performance and accessibility in these areas. Moderately high coverage was observed in Bono (80.8%), Eastern (78.8%), Central (76.7%), Ashanti (76%), and Ahafo (71.3%), while the Upper West (70%) and Upper East (70.3%) regions also performed reasonably well. In contrast, uptake was considerably lower in the Oti (72.5%), Bono East (62.5%), Northeast (52.3%), Northern (46.9%), and especially the Savannah (32.8%) regions, underscoring persistent inequities between the southern and northern belts of the country (see Fig. 1).

### Inequalities in HIV testing uptake among pregnant women during antenatal care in Ghana by education, marital status, and place of residence

**Fig. 2:**
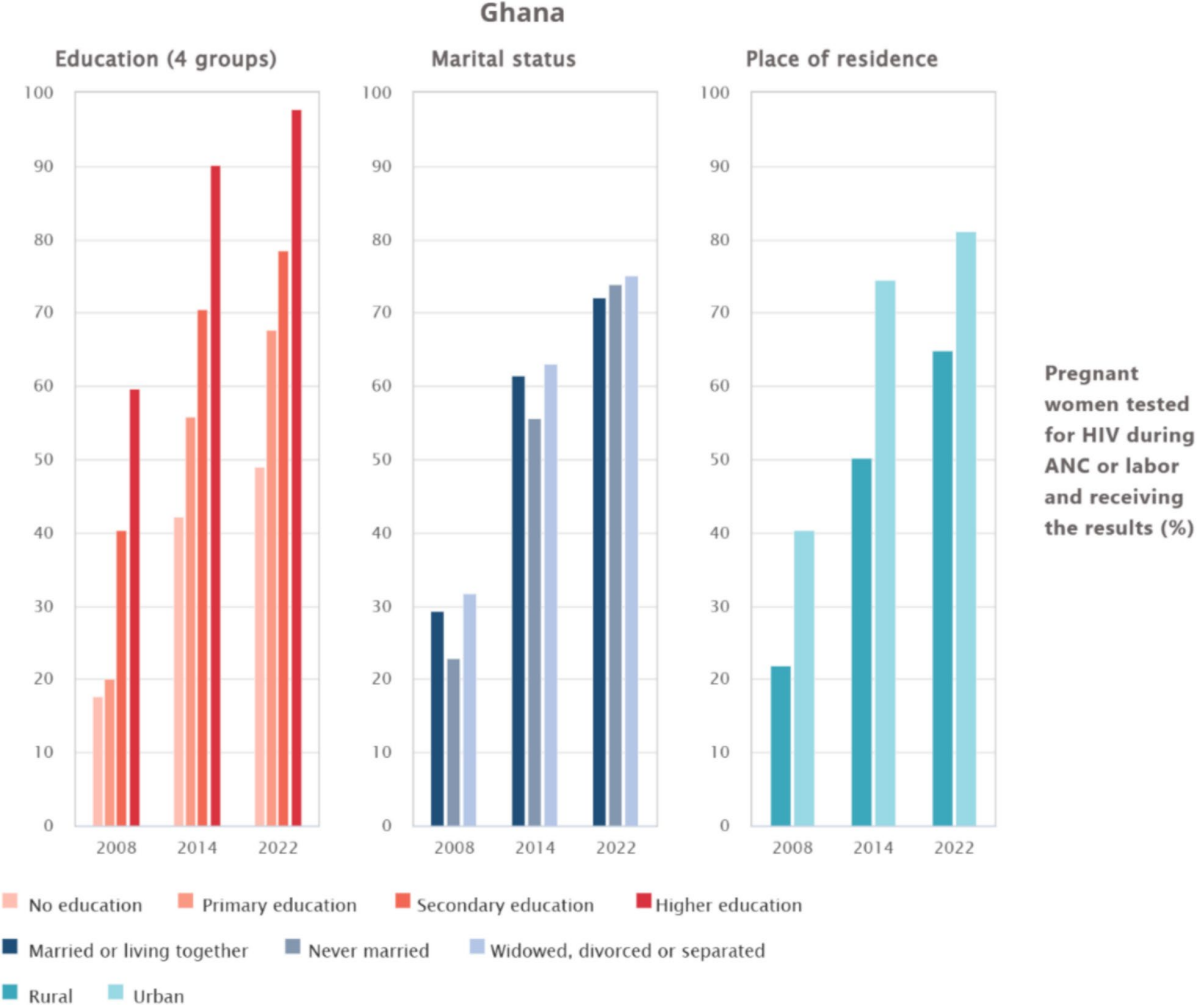
Inequalities in HIV testing uptake among pregnant women during antenatal care in Ghana by education, marital status, and place of residence

The analysis of HIV testing uptake among pregnant women in Ghana from 2008 to 2022 shows steady improvements across education, marital status, and place of residence. For education, uptake among women with no education was about 18% in 2008 but rose to nearly 60% in 2014 and further above 60% in 2022, while those with primary education increased from roughly 40% in 2008 to over 70% in 2014 and about 80% in 2022. Women with secondary education recorded higher progress, rising from nearly 60% in 2008 to about 90% in 2014 and above 90% in 2022, whereas those with higher education consistently had the greatest uptake, increasing from about 60% in 2008 to 90% in 2014 and almost 100% in 2022. For marital status, women married or living together had higher coverage, rising from around 25% in 2008 to above 70% in 2022, while never-married women increased from about 20% in 2008 to nearly 75% in 2022; widowed, divorced, or separated women also improved steadily, from around 30% in 2008 to over 70% in 2022. Place of residence showed persistent gaps, with rural women rising from about 20% in 2008 to nearly 70% in 2022, compared with urban women who increased from 40% to over 80% within the same period (see Fig. 2).

### Inequalities in HIV testing uptake among pregnant women during antenatal care in Ghana by age and economic status

**Fig. 3:**
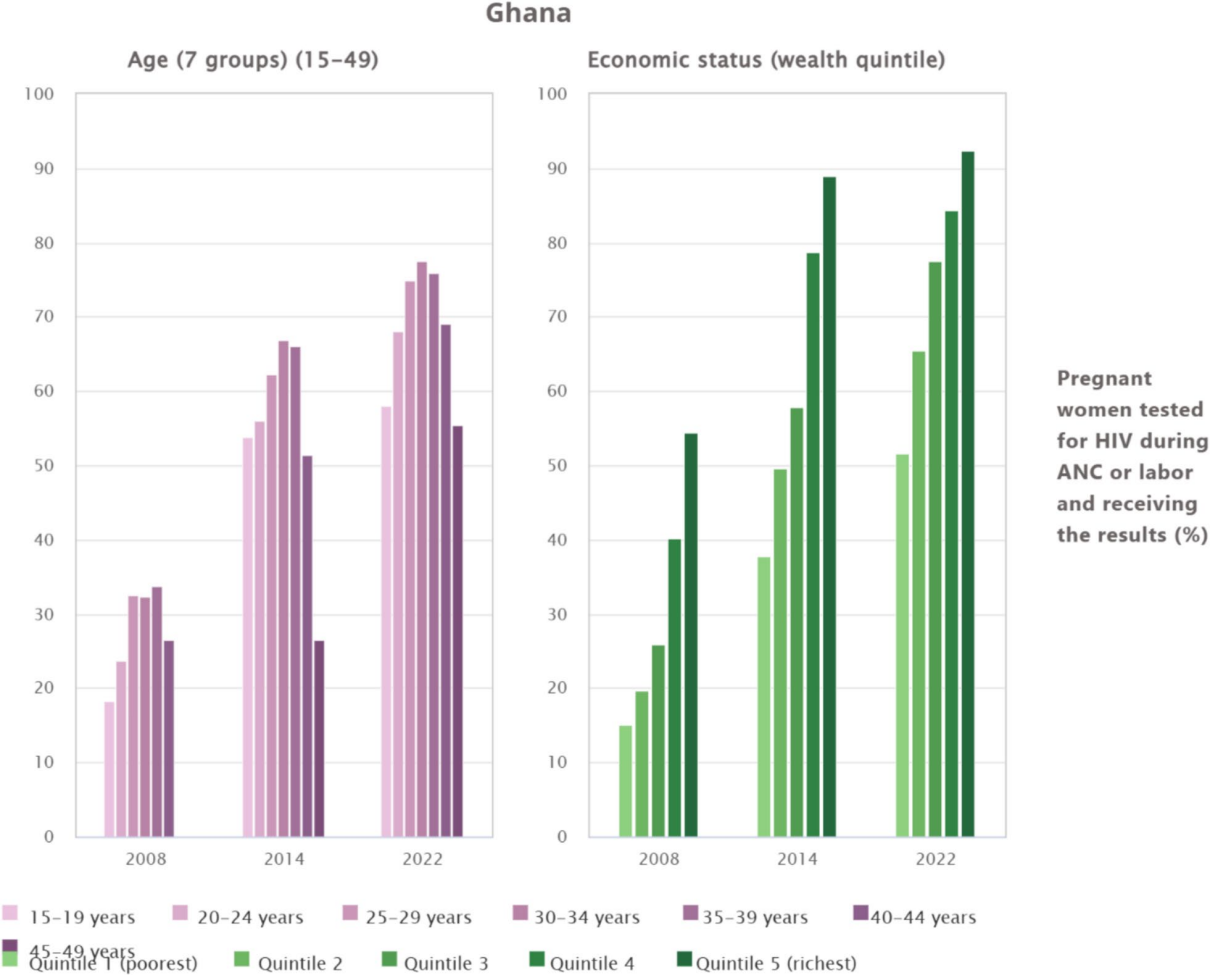
Inequalities in HIV testing uptake among pregnant women during antenatal care in Ghana by age and economic status

Between 2008 and 2022, Ghana recorded substantial progress in HIV testing among pregnant women, with overall coverage improving across both age and wealth groups. Nonetheless, marked disparities persist. By age, women in their peak reproductive years (25–39 years) consistently showed the highest uptake, with rates exceeding 74% in 2022, whereas adolescents (15–19 years) and older women (45–49 years) lagged at 58.1% and 55.5% respectively. By economic status, a persistent wealth gradient was evident, while the richest quintile reached 92.3% in 2022, only 51.7% of the poorest quintile accessed testing, despite notable gains since 2008 (see Fig. 3).

### Trends and socio-demographic inequalities in HIV testing uptake among pregnant women in Ghana, 2008–2022

The decomposition analysis of HIV testing uptake among pregnant women in Ghana between 2008 and 2022 indicates substantial national expansion in coverage, increasing from 28.9% in 2008 to 61.0% in 2014 and 72.4% in 2022. Age-related inequality was minimal. The ACI was small and statistically non-significant, increasing slightly from 0.9% (95% CI − 2.2 to 4.1) in 2014 to 1.8% (95% CI − 0.6 to 4.2) in 2022. The PAF values remained at 0.0% across survey years, indicating that age was not a major contributor to inequality. By contrast, economic status showed pronounced and persistent disparities. The ACI increased from 7.4% (95% CI 4.9–9.9) in 2008 to 10.5% (95% CI 8.6–12.3) in 2014, before declining to 8.2% (95% CI 7.2–9.1) in 2022. This decline reflects a partial flattening of the socioeconomic gradient as overall coverage expanded, rather than the elimination of wealth-based inequities. Absolute gaps remained substantial, with testing uptake among the poorest women still slightly above 50% in 2022. The PAF declined from 87.9% (95% CI: 87.7–88.2) in 2008 to 27.5% (95% CI 27.4–27.5) in 2022, indicating reduced relative concentration but not equitable coverage. Also, educational inequalities persisted across survey years. The ACI increased from 6.1% (95% CI 3.2–9.0) in 2008 to 7.2% (95% CI 6.1–8.3) in 2014 and remained at 7.2% (95% CI 5.1–9.4) in 2022. Although the PAF declined markedly from 106.0% (95% CI 105.4–106.5) in 2008 to 34.8% (95% CI 34.8–34.9) in 2022, sizeable absolute differences between women with no education and those with secondary or higher education remained. Further, marital status contributed comparatively little to overall inequality. Absolute differences decreased from 8.9% in 2008 to 3.1% in 2022, with corresponding reductions in PAF from 9.8% (95% CI 9.4–10.3) to 3.8% (95% CI 3.7–3.9). Rural–urban disparities persisted throughout the study period. The absolute difference peaked at 24.3% in 2014 and declined to 16.4% in 2022. Despite this improvement, rural women continued to experience lower coverage, and the PAF remained non-trivial at 12.1% in 2022. Ultimately, subnational variation was the most pronounced and persistent source of inequality. Absolute regional differences increased from 39.7% in 2008 to 56.1% in 2022, indicating widening gaps between the best- and worst-performing regions. Although the PAF declined from 70.1% (95% CI 69.8–70.3) to 22.7% (95% CI 22.7–22.8), substantial absolute regional disparities remained. These findings underscore that improvements in national coverage have not translated into equitable access, particularly across geographic regions (see Table 1).

**Table 1:** Trends and socio-demographic inequalities in HIV testing uptake among pregnant women in Ghana, 2008–2022

Dimension	N	Measure	2008 (28.9%)			2014 (61%)			2022 (72.4%)		
			Estimate (%)	CI-LB	CI-UB	Estimate (%)	CI-LB	CI-UB	Estimate (%)	CI-LB	CI-UB
*Age (7 groups) (15–49)*	6915	ACI	NA	NA	NA	0.9	− 2.2	4.1	1.8	− 0.6	4.2
		D	NA	NA	NA	− 27.3	NA	NA	− 2.6	NA	NA
		PAF	0.0	− 0.3	0.3	0.0	− 0.2	0.2	0.0	− 0.2	0.2
		PAR	0.0	− 10.2	10.2	0.0	− 14.8	14.8	0.0	− 13.7	13.7
		R	NA	NA	NA	0.5	NA	NA	1.0	NA	NA
*Economic Status (Wealth Quintile)*	6932	ACI	7.4	4.9	9.9	10.5	8.6	12.3	8.2	7.2	9.1
		D	39.3	NA	NA	51.2	NA	NA	40.6	NA	NA
		PAF	87.9	87.7	88.2	45.8	45.7	45.8	27.5	27.4	27.5
		PAR	25.5	18.4	32.5	27.9	24.3	31.6	19.9	17.4	22.4
		R	3.6	NA	NA	2.4	NA	NA	1.8	NA	NA
*Education (4 groups)*	6932	ACI	6.1	3.2	9.0	7.2	6.1	8.3	7.2	5.1	9.4
		D	42.0	NA	NA	48.0	NA	NA	48.8	NA	NA
		PAF	106.0	105.4	106.5	47.7	47.6	47.8	34.8	34.8	34.9
		PAR	30.7	14.0	47.3	29.1	23.0	35.2	25.2	22.8	27.7
		R	3.4	NA	NA	2.1	NA	NA	2.0	NA	NA
*Marital status*	6931	D	8.9	NA	NA	7.5	NA	NA	3.1	NA	NA
		PAF	9.8	9.4	10.3	3.4	3.2	3.5	3.8	3.7	3.9
		PAR	2.8	− 10.6	16.3	2.1	− 7.5	11.7	2.7	− 3.9	9.3
		R	1.4	NA	NA	1.1	NA	NA	1.0	NA	NA
*Place of residence*	6933	D	18.6	NA	NA	24.3	NA	NA	16.4	NA	NA
		PAF	39.4	39.3	39.5	22.1	22.0	22.1	12.1	12.1	12.1
		PAR	11.4	8.0	14.8	13.5	11.2	15.8	8.8	7.2	10.4
		R	1.9	NA	NA	1.5	NA	NA	1.3	NA	NA
*Subnational region*	6931	D	39.7	NA	NA	53.8	NA	NA	56.1	NA	NA
		PAF	70.1	69.8	70.3	34.2	34.1	34.3	22.7	22.7	22.8
		PAR	20.3	12.3	28.3	20.9	16.7	25.1	16.5	11.0	21.9
		R	5.2	NA	NA	2.9	NA	NA	2.7	NA	NA

D = absolute difference between most-and least-advantaged subgroups; R = ratio; ACI = absolute concentration index (× 100; negative = pro-poor); PAR = population attributable risk (percentage points); PAF = population attributable fraction (%), bounded between − 100% and + 100. All estimates are weighted; 95% CIs reflect complex survey design

## Discussion

The study examined trends and inequalities in HIV testing uptake among pregnant women in Ghana between 2008 and 2022. Findings indicate a significant improvement in coverage, rising from 28.9% in 2008 to 72.4% in 2022. These results are consistent with previous Ghanaian studies that documented improvements in HIV testing following the expansion of prevention of mother-to-child transmission services [25, 26]. The study also supports global estimates reporting increasing uptake of antenatal HIV testing across sub-Saharan Africa [16]. These gains reflect the successful institutionalisation of routine HIV testing within antenatal and delivery care. However, overall progress masks persistent and statistically significant inequalities across population subgroups.

Regional disparities were the most pronounced and consistent source of inequality. Near-universal uptake in Volta and Greater Accra contrasts sharply with substantially lower coverage in Savannah and Northern regions. These differences are not merely descriptive. Inequality measures with confidence intervals excluding the null indicate statistically significant regional gradients. Such patterns point to structural determinants, including unequal distribution of health facilities, shortages of skilled health personnel, weaker referral systems, and logistical constraints affecting test kit availability in northern Ghana. These findings align with prior evidence on geographic inequities in maternal health service capacity and utilisation [27]. Also, socioeconomic inequalities remained evident. Women in the richest wealth quintile had markedly higher testing uptake than those in the poorest quintile. While reductions in concentration indices and population attributable fractions suggest a relative narrowing of wealth-related inequality over time, these metrics should be interpreted as descriptive indicators of redistribution rather than causal effects. The persistence of absolute coverage gaps indicates that financial constraints, transport costs, and opportunity costs of care-seeking continue to limit access among poorer households. These findings are consistent with the literature linking economic disadvantage to reduced utilisation of maternal and HIV services [4, 28]. Nonetheless, the observed decline in wealth-related concentration indices and population attributable fractions suggests that economic disparities are narrowing as national coverage expands, a pattern supported by evidence from other African countries where rapid programme scale-up has reduced inequalities [29, 30].

Educational attainment emerged as another key determinant. Uptake among women with higher education was nearly universal by 2022, compared with slower progress among those without formal schooling. This result is in line with studies linking maternal education to better health literacy and greater utilisation of antenatal and HIV services [31, 32]. Similarly, rural–urban disparities have reduced over time, reflecting gains from outreach and facility-based interventions, although urban women still maintain an advantage [21]. Again, age differences were less marked in the inequality decomposition, although adolescents and older women remained less likely to test compared with women in their peak reproductive years. This finding is consistent with studies highlighting stigma, low risk perception, and limited decision-making power among these groups [33, 34]. Marital status was only marginally associated with inequality, but testing was more common among married women, echoing evidence that partner support encourages HIV service uptake [35–37]. Ultimately, the findings indicate that Ghana’s progress in antenatal HIV testing has been substantial but uneven. Structural barriers linked to health system capacity and geographic access coexist with demand-side constraints shaped by socioeconomic position, education, and social norms. Policy responses should therefore be differentiated. Supply-side interventions must prioritise under-resourced regions through targeted investment in infrastructure, workforce deployment, and supply chain reliability. Concurrently, demand-side strategies should address stigma, health literacy, and community engagement, particularly among poorer, less-educated, and rural populations. Addressing both dimensions is essential for sustaining gains and achieving equitable elimination of mother-to-child transmission of HIV.

### Strengths and limitations

A key strength of this study lies in its use of nationally representative DHS data spanning over a decade, allowing robust temporal comparisons of HIV testing uptake among pregnant women in Ghana. The DHS employs standardised methodologies across survey waves, ensuring consistency in data collection and enhancing the reliability of observed trends. The inclusion of multiple equity stratifiers such as education, marital status, place of residence, region, age, and wealth status enabled a nuanced decomposition analysis, highlighting sources of inequality across social and demographic subgroups. Moreover, the decomposition of ACI, D, and PAF provided a comprehensive view of disparities and their evolution over time, offering policy-relevant insights. The large sample size further strengthened the statistical power and precision of the estimates, permitting stratified analyses across subpopulations. However, certain limitations must be acknowledged. First, the cross-sectional design of DHS data restricts causal inference, as associations observed cannot establish temporal or directional relationships. Self-reported measures of HIV testing may be subject to recall or social desirability bias, potentially inflating coverage estimates. Regional disparities, while highlighted, may mask within-region heterogeneities that are not fully captured by the dataset. Although decomposition analysis illuminates inequalities, it cannot fully account for unobserved confounders such as stigma, health system quality, or cultural norms that may influence testing behaviours. Additionally, the HEAT-based decomposition measures do not adjust for covariates and may be sensitive to administrative boundary changes, which could influence the magnitude of observed inequalities. Furthermore, the reliance on household survey data excludes women outside the survey frame, such as those without permanent residence, potentially under-representing highly vulnerable populations. Finally, while DHS provides valuable quantitative insights, it lacks qualitative dimensions that could contextualise barriers and facilitators of HIV testing among pregnant women.

### Implications for policy, practice, and research

Persisting regional and socioeconomic disparities in HIV testing among pregnant women highlight areas where equity monitoring should inform policy attention, rather than implying specific causal failures. The findings suggest that subnational regions with consistently lower coverage, particularly Savannah, Northern, and North East, warrant closer policy focus. Within existing national strategies, the Ministry of Health (MoH) and the Ghana Health Service may use these results to guide prioritisation of monitoring, planning, and resource distribution. Development partners, including UNAIDS, the World Health Organization, and UNICEF, may also draw on these equity patterns to support geographically targeted programme reviews within the broader goals of Universal Health Coverage (UHC) and Sustainable Development Goal 3.

Frontline health workers, including midwives, community health nurses, and antenatal care providers, remain central to ensuring consistent delivery of HIV testing across settings. Regional and district health directorates may use routine data to identify persistent gaps and support supervision and service monitoring. Community-level engagement, involving community health workers and local opinion leaders, may be considered to support demand for testing in underserved populations. These implications are offered as programmatic considerations, not as evidence of specific behavioural or system-level determinants.

From a research perspective, the observed disparities underscore the need for analytical studies beyond descriptive inequality monitoring. Future work using multivariable approaches is required to examine the relative contributions of socioeconomic position, service accessibility, stigma, and health system factors. Qualitative and mixed-methods studies would be particularly valuable in low-performing regions to contextualise observed patterns. Further research evaluating alternative service delivery models, including outreach and integrated maternal–child health services, could provide empirical evidence to inform targeted interventions. Such work would complement equity surveillance and strengthen the evidence base for reducing persistent gaps in HIV testing among pregnant women in Ghana.

## Conclusion

The analysis demonstrates substantial progress in HIV testing uptake among pregnant women in Ghana, with coverage increasing from less than one-third in 2008 to nearly three-quarters in 2022, reflecting the expansion of antenatal care services and sustained national policy commitment. Improvements were observed across education, marital status, wealth, and place of residence. However, pronounced inequalities persist, with subnational regions remaining the dominant source of disparity, as southern regions such as Volta and Greater Accra approach universal coverage while northern regions, particularly Savannah, continue to lag markedly. Although wealth- and education-related gaps have narrowed, women from the poorest households, those without formal education, and rural residents remain disadvantaged, and adolescents and older women consistently show lower uptake than women in their prime reproductive years. Ultimately, despite notable national gains, targeted equity-focused strategies are required to address persistent regional and socio-economic inequalities, strengthen rural health system capacity, and ensure equitable access to HIV testing and timely results for all pregnant women.

## Data Availability

The dataset used can be accessed at https://www.who.int/data/inequality-monitor/assessment_toolkit.

## Abbreviations

ACI: Absolute Concentration Index
ANC: Antenatal care
CI: Confidence interval
D: Difference
DHS: Demographic and health survey
GHS: Ghana health service
GSS: Ghana statistical service
HIV: Human Immunodeficiency virus
MoH: Ministry of Health
PAF: Population attributable fraction
PAR: Population attributable risk
PMTCT: Prevention of mother-to-child transmission
R: Ratio
SDG: Sustainable development goal
SSA: Sub-Saharan Africa
UNAIDS: Joint United Nations Programme on HIV/AIDS
UNICEF: United Nations Children’s Fund
UHC: Universal Health Coverage
WHO: World Health Organization
